# Cytology Smears: An Enhanced Alternative Method for Colorectal Cancer pN Stage—A Multicentre Study

**DOI:** 10.3390/cancers14246072

**Published:** 2022-12-09

**Authors:** Sherley Diaz-Mercedes, Ivan Archilla, Sara Lahoz, Maria Teresa Rodrigo-Calvo, Sandra Lopez-Prades, Jordi Tarragona, Stefania Landolfi, Angel Concha, Isidro Machado, Joan Maurel, Nuria Chic, Antoni Castells, Francesc Balaguer, Jordi Camps, Miriam Cuatrecasas

**Affiliations:** 1 Department of Pathology, Hospital Clinic of Barcelona, University of Barcelona, 08036 Barcelona, Spain; 2 Institut d’Investigacions Biomèdiques August Pi i Sunyer (IDIBAPS), Hospital Clinic of Barcelona, 08036 Barcelona, Spain; 3 Gastroenterology Department, Hospital Clinic of Barcelona, 08036 Barcelona, Spain; 4 Pathology Department, Hospital Arnau de Vilanova, 25198 Lleida, Spain; 5 Pathology Department, Vall de Hebron University Hospital, 08035 Barcelona, Spain; 6 Department of Pathology, University Hospital A Coruña, 15006 A Coruña, Spain; 7 Pathology Department, Instituto Valenciano de Oncologia, 46009 Valencia, Spain; 8 Pathology Laboratory, Hospital Quiron Salud, 46009 Valencia, Spain; 9 Oncology Department, Hospital Clinic of Barcelona, 08036 Barcelona, Spain; 10 Centro de Investigación Biomédica en Red en Enfermedades Hepáticas y Digestivas (CIBEREHD), University of Barcelona, 08036 Barcelona, Spain; 11 Basic Clinical Practice Departament, University of Barcelona, 08036 Barcelona, Spain; 12 Medicine Department, University of Barcelona, 08036 Barcelona, Spain; 13 Department of Cell Biology, Physiology and Immunology, Faculty of Medicine, University Autonomous of Barcelona, 08193 Bellaterra, Spain

**Keywords:** colorectal cancer, lymph node, staging, diagnosis, cytology, OSNA

## Abstract

**Simple Summary:**

Recurrence of stage II (pT3-T4 pN0) colorectal cancer (CRC) occurs in about 15% of patients and it is often due to undetected lymph node (LN) metastases with conventional pathology haematoxylin and eosin (H&E) LN analysis. Despite more sensitive molecular methods of LN staging having proved to have prognostic value in stage II CRC, we aimed at determining whether the pN stage could be better assessed with LN cytology smears. We analysed 3936 LNs from 217 CRC surgical resections, using three methods, H&E, cytology smears, and the One Step Nucleic Acid Amplification (OSNA) molecular assay. We compared the pN stages obtained from both H&E and cytology, as well as with the OSNA results. We concluded that LN analysis with cytology smears not only enables performing the pN stage, but detects more LN metastases than H&E, with a similar detection rate to molecular methods. Cytology LN analysis would allow a better patient therapeutic management.

**Abstract:**

Stage II colorectal cancer (CRC) recurrence remains a clinical problem. Some of these patients are true stage III CRC with a pN0 pathology stage. This large prospective multicentre cohort study aimed at evaluating the diagnostic ability of lymph node (LN) cytology smears to perform the pN stage and compare it with the conventional haematoxylin and eosin (H&E) pathology pN stage. Additionally, we used the One-Step Nucleic Acid Amplification (OSNA), a high-sensitive molecular method of LN staging. A total of 3936 fresh LNs from 217 CRC surgical specimens were examined by three methods, H&E, LN cytology smears, and OSNA. H&E detected 29% of patients with positive LNs, cytology smears 35%, and OSNA 33.2% (*p* < 0.0001). H&E and cytology concordantly classified 92.2% of tumours, and 88.5% between OSNA and H&E. Cytology had 96.8% sensitivity and 90.3% specificity to discriminate positive/negative patients compared to H&E (*p* = 0.004), and 87.3% sensitivity and 89% specificity when compared to OSNA (*p* = 0.56). Patients with positive LNs detected by any of the three methods had significantly worse disease-free and overall survival. We conclude that pN stage accuracy for detecting positive LNs is superior with LN cytological smears than with conventional H&E, which would enable a better pN stage and management of early-stage CRC patients.

## 1. Introduction

The presence of lymph node (LN) metastases greatly influences colorectal cancer (CRC) patients’ survival at any T stage of the disease, as it determines the need for adjuvant chemotherapy [1,2,3]. According to current CRC diagnostic protocols, the LN stage (pN) is based on the number of positive formalin-fixed paraffin-embedded (FFPE) LNs detected on routine haematoxylin and eosin (H&E) stains [2]. Yet, the landscape of CRC has recently changed with the introduction of population-based screening programs, which encompass an increased diagnosis of patients at earlier stages of the disease. In this new scenery, stages I-II (T1-T4 N0) CRC represent 70% of all CRC diagnoses [4,5,6,7,8]. Furthermore, in the early stages of CRC, LN metastases are often small sized, thus, H&E LN analysis could oversight the presence of micrometastases, since only a small part of the LN is examined [9]. In fact, the main reason for the low sensitivity of H&E pN staging is tissue allocation bias. Still, this deficiency is well known. Therefore, pathology diagnosis guidelines require the analysis of a minimum of 12 LNs for a reliable pN0 stage [10,11]. 

On the one hand, in the new arena of CRC screening, there is an unmet clinical need to incorporate more sensitive, reliable, and efficient diagnostic methods for a more accurate pN stage, especially for stage II CRC, which would allow better clinical management of these patients [9,12]. On the other hand, the use of molecular methods of LN analysis allows for the identification of patients at risk of progression, not detected by conventional H&E [9,13,14]. In fact, it is well established that molecular detection of micrometastases, in otherwise H&E negative LNs from CRC surgical specimens, has been associated with worse prognosis [9,12,15]. The One-Step Nucleic Acid Amplification (OSNA) molecular assay is a quantitative, fast, and reproducible RT-PCR-based method for the detection of cytokeratin 19 (CK19) mRNA. It has been validated for the analysis of sentinel LNs in breast carcinoma and for CRC pN stage [12,16,17,18,19,20,21,22,23,24]. Its results are expressed as the total tumour load (TTL), defined as the amount of CK19 mRNA copies/µL present within all the LNs analysed from a surgical specimen [17,21]. In breast carcinoma, molecular LN assessment gives information on the amount of tumour burden present in the LN compartment, which has predictive and prognostic values [16,17,18,19,20,21]. In CRC, the TTL has prognostic value [12,15]. 

Diagnostic cytology is the science of diagnosis of disease through the analysis of cells. It was introduced in 1928 by George N Papanicolaou as a tool to detect cancer and precursor lesions. It has been widely used in cervical and anal cancer screening programs, which have achieved a great reduction in its incidence in many countries [25,26]. This simple morphology-based diagnostic method is routinely used in clinical practice for the diagnosis, decision-making, and treatment of benign and malignant conditions, either alone or complementary to standard surgical pathology diagnosis [27,28,29]. Diagnostic cytology has many advantages, such as being a simpler and less-invasive procedure than biopsy or surgical resection. It is also inexpensive and has a fast turnaround time for diagnosis reporting. 

In this large prospective multicentre cohort study on CRC, we aimed to evaluate the diagnostic ability of cytology-based LN analysis to perform the pN stage and compare it to the standard H&E pN stage, as well as to the high-sensitive OSNA molecular method. We demonstrated the capability of performing the pN stage with cytology smears, and its superiority to detect positive LNs compared to conventional H&E, with a similar detection rate to molecular methods. The use of cytology smears would enable to obtaina more accurate pN stage in CRC, enabling a better patient therapeutic management, and at the same time the use of the whole LN tissue for molecular analysis. 

## 2. Materials and Methods

### 2.1. Patients and Inclusion Criteria

This is a large observational and prospective multicentre cohort study conducted from November 2016 to December 2019 at five tertiary university hospitals. The study was approved by the ethic and scientific committees of all centres (Reg. 2012/7324) and all patients signed the informed consent to participate in the study. All CRC patients undergoing curative-intended surgery were considered for the study. Inclusion criteria were patients over 18 years-old, with primary histologically confirmed CRC, positive for cytokeratin 19 (CK19) immunohistochemistry (IHC). Exclusion criteria were patients with non-invasive tumours, synchronous carcinomas, metastatic carcinomas, presence of neoadjuvant or adjuvant therapy, familial adenomatous polyposis syndrome, carcinomas on inflammatory bowel disease, stent-type intraluminal devices or presence of other malignancies.

### 2.2. Fresh Lymph Node Dissection and LN Analysis

**Figure 1:**
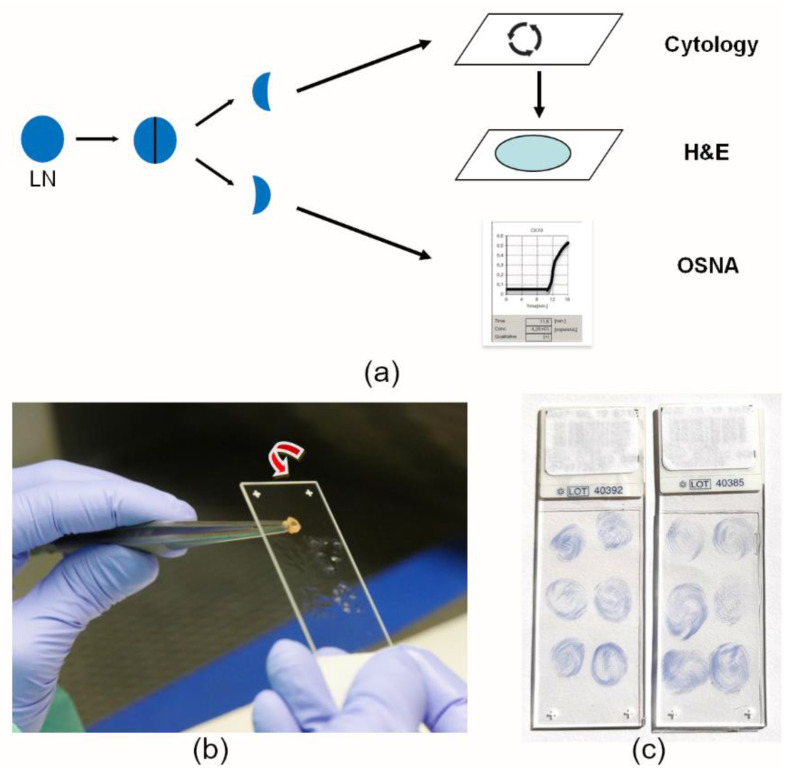
Fresh lymph node analysis with three methods, cytology smears, H&E, and OSNA. (**a**) Dissected lymph nodes were bisected. A cytology smear was performed on a pre-treated slide, and that part of the lymph node was then used for FFPE and histology analysis with H&E. The other half of the LN was used for the molecular OSNA assay. (**b**) For each LN, cytology smears were performed by making a circular movement with gentle pressure on the pre-treated slide to ensure a smooth monolayer of cells. (**c**) Slides containing six lymph node smears were air-dried and immunostained with CK19 antibody, using haematoxylin as counterstain.

Surgical specimens from CRC were received at the pathology department immediately after surgical excision and fresh LN dissection was performed within 45 min of surgical extraction. Freshly dissected LNs were bi-sectioned. One-half of the LN was used to perform cytology smears by making a circular movement on the surface of a pre-treated slide. Then, that half of the LN was submitted for conventional FFPE tissue processing. The other half of the LN was placed into a microcentrifuge tube for deferred OSNA analysis, which was performed using the pooling method, as described in Rakislova et al. [30] (Figure 1a,b). The same procedure was performed with each LN. Each slide contained smears from six LNs. Slides were air-dried and CK19-IHC staining was performed either on the same day or stored at −20 °C for further immunostaining on subsequent days. After the IHC staining was performed on the cytology smears, the slides were ready for microscopic assessment and cytology pN staging (Figure 1c). After LN dissection, the surgical specimen was routinely processed following overnight formalin fixation. In some cases, a few additional LNs were observed on H&E slides, located in the fat by the colorectal wall. These FFPE LNs were only considered for the H&E pN stage since fresh analysis by cytology or OSNA was not performed.

### 2.3. One-Step Nucleic Acid Amplification (OSNA) Assay

The OSNA assay is a simple, standardized, and fast process for LN analysis. It is an automated molecular method based on the quantification of the amount of cytokeratin 19 mRNA, performed with the RD-100i system (Sysmex, Kobe, Japan). It uses the RT-LAMP, an RT LOOP-mediated isothermal amplification at 65 °C. In contrast to RT-PCR, there is no need for mRNA extraction or purification. LN tissue is homogenized in a specific lysis buffer and centrifugated, then, the lysate supernatant is used for amplification. Quantification is performed by the detection of precipitated results in turbidity of the magnesium pyrophosphate, a by-product of the amplification process. The system is run with adequate controls, i.e., b-actin mRNA to check mRNA quality, a positive control with a given number of copies of CK19 mRNA, and a negative control without CK19 mRNA, which are used for calibration and contamination check. Cross-amplification with the two CK19 pseudogene products is prevented by using six primers, including the forward and reverse loop primers, thus, avoiding simultaneous genomic DNA amplification [31,32]. 

The molecular LN assessment with the OSNA assay was performed according to the manufacturer’s instructions using the pooling method, in which each PCR tube contained multiple LNs from the mesocolon or mesorectum, as described in Rakislova et al. [30]. The OSNA results were obtained in 20 to 40 min and were expressed as the total tumour load (TTL), defined as the tumour burden contained in all LNs analysed from a given case. The TTL was considered positive when values were ≥250 copies/μL [32,33].

### 2.4. CK19 Immunochemistry

**Figure 2:**
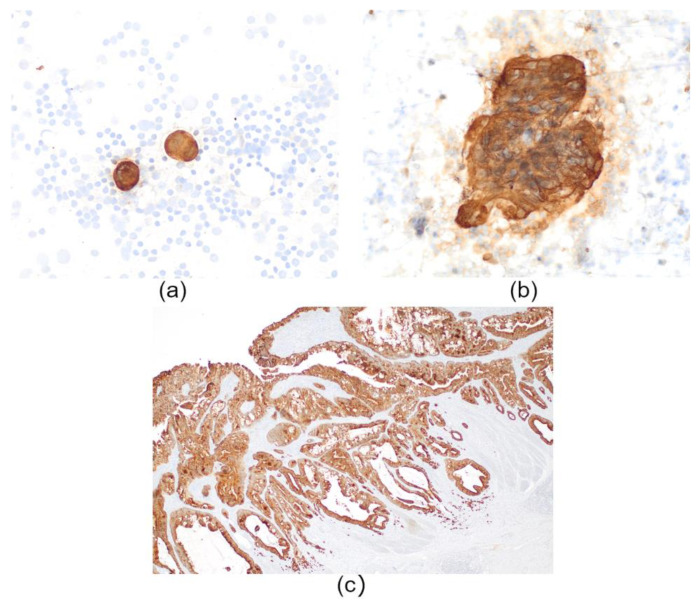
CK19 immunochemistry. (**a**) Positive CK19 immunocytochemistry stains of cytology smears from different lymph nodes showing few solitary tumour cells. (**b**) A group of positive tumour cells from a cytology smear. (**c**) A primary colorectal carcinoma positive for CK19 immunohistochemistry.

The slides containing LN smears were immersed in absolute alcohol for 10 min and a standard immunocytochemistry protocol was performed, without the need for antigen retrieval. CK19 immunostains were performed using the Autostainer Link 48 (Agilent, Santa Clara, CA, United States), with 20 min incubation with the primary CK19 antibody (CK19 mouse monoclonal, clone RCK108; IR615 pre-diluted. Agilent, Santa Clara, CA, United States). In cytology smears, membranous staining with or without cytoplasm staining of tumour epithelial cells, either solitary or in groups, was considered positive (Figure 2a,b). Immunohistochemistry was also performed on all primary tumours. This was performed to ensure that all tumours were positive for CK19. Then, a negative OSNA LN analysis could be considered a real negative, and not due to the negativity of the primary tumour for CK19. The standard CK19 IHC protocol was used as described in Aldecoa et al. [14]. Staining of at least 10% of the primary tumour was considered positive (Figure 2c).

### 2.5. Lymph Node Pathology Reporting and pN Staging

All freshly dissected LNs were analysed by three methods: (1) H&E; (2) LN cytology smears immunostained with CK19; and (3) the OSNA assay (Figure 1a) by four gastrointestinal pathologists (SDM, IA, MTR, and MC). The pN stage of the pathology report resulted from the standard H&E histological diagnosis, performed according to the pTNM classification of the AJCC, 8th Edition [10]. The pN stage evaluation from cytology smears was as follows; each slide contained smears from six LNs. Each smear was individually analysed. Any CK19 IHQ-positive epithelial cells, either individually or in groups, were considered positive. Then, the final number of positive smears corresponded to the number of positive LNs, which was converted into the pN cytology stage. Any information obtained from the pN stage from cytology smears or the TTL from the OSNA assay was not included in the final pathology report and was blind to the pathologist and clinician, as they were assessed after the regular pathology report was issued.

### 2.6. Statistical Analysis

All analyses were carried out using R statistical language version 3.6. Numerical variables were tested for their normal distribution using Shapiro-Wilk’s statistics. To test the association of numerical variables between groups, Student’s *t*-test was applied for parametric data and the Mann-Whitney-Wilcoxon U test for non-parametric. Fisher’s exact test was utilized to calculate *p*-values in the case of categorical classes. Correlation scores regarding the number of positive LNs between methods were calculated using Spearman’s rank test. Correlation scores regarding nominal pN stages (pN0, pN1, pN2) were evaluated with Cohen’s kappa statistics in the fmsb package. Classification performance of cytological smears to discriminate LN-positive patients in comparison to H&E and OSNA was calculated using caret package in terms of sensitivity and positive predictive value (PPV), and to discriminate LN-negative cases in terms of specificity and negative predictive value (NPV). McNemar’s chi-squared test was utilized to assess the symmetry of paired nominal data in a two-dimensional contingency table.

### 2.7. Study Endpoints and Survival Analysis 

The positive detection rate (PDR) of each method of LN analysis was considered the study’s primary endpoint. PDR was defined as the proportion of patients with positive LNs. Disease-free survival (DFS) and overall survival (OS) were assessed as secondary study endpoints. DFS was defined as the time from surgical intervention to relapse or death, whichever occurred first. OS was the time from surgical intervention to patient death. Data for patients who experienced no event were censored at the time of the last follow-up. The Kaplan–Meier method in the survival R package was applied to estimate time-to-event values, and a log-rank test was used to test statistical significance in DFS and OS between patient arms. Hazard ratios (HR) and their associated 95% confidence intervals (CI) were inferred with the use of a Cox regression model with proportional hazards in a univariate and multivariable manner. Variables used for adjusting multivariable models were patient age, sex, pT stage, and histological grade. 

## 3. Results

### 3.1. Clinical and Pathological Characteristics

A total of 217 CRC patients were prospectively included in this observational multicentre cohort study. Clinical and pathological characteristics of the patients and tumours are shown in Table 1. Patients’ median age at surgery was 71 years-old (range 39–92) and 59.4% were male. Most tumours (71%) were stage I-II. A median of 18 LNs were dissected per patient (range 4–62), being 17 of them freshly isolated. A total of 4310 LNs from 217 CRC surgical specimens were isolated, of which 3936 (91.3%) were freshly acquired. Post-formalin-fixation LNs were observed in the fat tissue adjacent to the colorectal wall. 

### 3.2. Patient Positive Detection Rate Is Higher with Cytology Smears and OSNA Than with H&E

**Figure 3:**
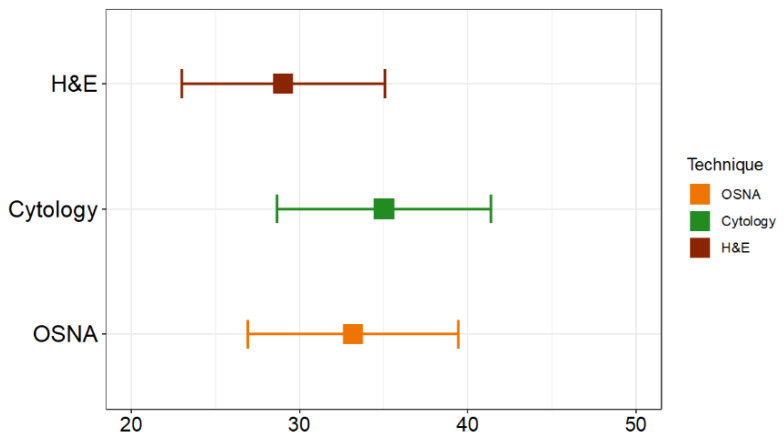
Forest plot displaying the positive detection rate, or proportion of patients with positive lymph nodes, of the three methods of LN analysis. H&E: haematoxylin and eosin; OSNA: one-step nucleic acid amplification.

The average positive detection rate (PDR) by the three methods was 70 patients (32.3%; 95% CI, 26–38.5%). The PDR achieved with H&E was 63 patients (29%; 95% CI, 23–35%), compared to a PDR of 76 patients detected with the cytology-based analysis (35%; 95% CI, 28.7–41.4%), (*p* < 0.0001 compared to H&E). Regarding the OSNA assay, its PDR was of 72 patients (33.2%; 95% CI, 26.9–39.4%) (*p* < 0.0001 compared to H&E), (Figure 3; Table 2).

When focusing on the number of positive LNs detected by H&E and by cytology smears, we observed that cytology detected a higher number of positive LNs than H&E. With the standard H&E pathology examination we found 210/3936 (5.3%) positive LNs, while cytology smears detected 249/3936 (6.3%) positive LNs (Fisher’s *p* = 0.067) (Table 2). The Spearman’s correlation score for the number of positive LNs between H&E and cytology smears was 85.5% (*p* < 0.0001). The mean number of positive LNs per patient detected by H&E was 0.97 (range, 0–17) and by cytology 1.15 (range, 0–17).

### 3.3. pN Upstaging with LN Cytology Smears with Respect to H&E and High Diagnostic Efficacy of Cytology Smears to Discriminate LN-Positive Patients

We next sought to compare the pN stages obtained from conventional H&E LN analysis and cytological smears. H&E classified 154 (71%) pN0 patients, 46 (21.2%) pN1, and 17 (7.8%) pN2. In contrast, LN smears identified 141 (65%) pN0 patients, 55 (25.3%) pN1, and 21 (9.7%) pN2. Cytology upstaging was 6%, with 4.1% of patients reclassified as pN1 and 1.9% as pN2 (Table 3).

We subsequently assessed the diagnostic efficacy of cytology smears and OSNA-based analysis to identify LN-positive patients as compared with gold-standard H&E. Examination of 3936 LNs from 217 patients revealed that 200 tumours (92.2%) were concordantly classified by cytology-based analysis and routine H&E stains, (kappa = 0.82, *p* < 0.004), (92.2% accuracy; 95% CI: 87.8–95.4%) (Table 3). In detail, out of 63 (29%), LN-positive patients detected with H&E, only two of them were classified as LN-negative by cytology, resulting in 96.8% sensitivity for cytology to distinguish pN-positive cases. In parallel, the positive predictive value (PPV) of cytology was 80.3%, indicating that among the 76 positive patients detected by cytology, 61 of them (80.3%) were positive according to H&E. On the other hand, out of a total of 154 cases (71%) detected as LN-negative by conventional H&E, 15 of them were positive by cytology, leading to 90.3% specificity to differentiate pN-negative individuals (Table 3). 

When assessing OSNA versus H&E results, the two methods exhibited a global concordance rate of 192/217 cases (88.5% accuracy; 95% CI: 86.5–90.4%) (Table 3). Specifically, the sensitivity achieved by OSNA to detect LN-positive cases was 87.3%, since 8/63 patients positive with H&E were negative for OSNA, resulting in a PPV of 76.4%. Regarding the identification of LN-negative cases, OSNA was 89% specific, with 17/154 positive cases which were negative with H&E (Table 3).

Assessment of cytology versus OSNA results resulted that the two methods exhibited a global concordance rate of 191/217 cases (88% accuracy; 95% CI: 86.06–89.98%). Specifically, out of the 76 individuals determined as LN-positive by cytology, 61 of them (80.3%) were also positive according to OSNA-based analysis, with 80.3% sensitivity and 84.7% PPV for the detection of LN-positive cases. As regards to the identification of LN-negative cases, cytological smears detected 141 cases as negative (65%) while OSNA identified 145 (66.8%) negative cases, with a specificity of 92.2% and NPV of 89.7%.

When comparing the three methods of LN analysis, 183/217 were concordantly classified accounting for LN-positive and LN-negative cases (84.3% accuracy). Out of these, 55/217 (25.3%) were categorized as positive, and 128/217 (59%) as negative by the three techniques. All patients with positive LNs obtained post-formalin fixation also had other freshly processed positive LNs identified by either H&E or LN smears (*n* = 16/217 patients (7,4%); representing 72/374 (18.9%) positive LNs.

### 3.4. The Total Tumour Load (TTL) Increases with the pN Stage

**Figure 4:**
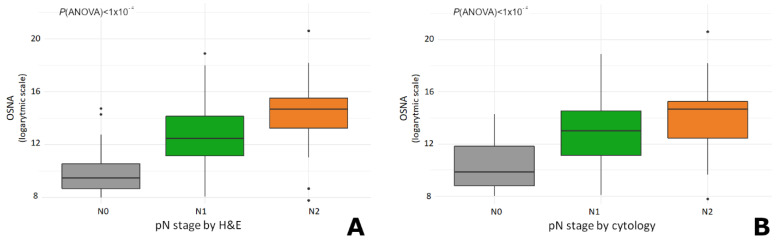
Total tumour load (TTL) values obtained by OSNA were correlative to the pN stages determined by (**A**) H&E and (**B**) cytology-based analysis. *p*-values were determined using an ANOVA test. Tumours with OSNA > 300,000 (superior outlier values) were excluded from both graphs to enable a better visualization between groups. H&E: haematoxylin and eosin; OSNA: one-step nucleic acid amplification.

We then compared the total tumour load (TTL) with the pN stages obtained by H&E and cytology-based LN analyses. In both assessments, the TTL significantly increased in accordance with the pN stage obtained by H&E (Figure 4a) and cytology (Figure 4b) (ANOVA’s *p* < 0.0001 in both analyses). The mean TTL values for the respective pN stages obtained by H&E were: 433.6 copies/μL for pN0 (range 0–27,300 copies/μL); 35,782.6 copies/μL for pN1 (range 0–488,000 copies/μL), and 144,651.8 copies/μL for pN2 cases (range 0–1,600,000 copies/μL). Similarly, the mean TTL values for the pN stages obtained by cytology were 253.7 copies/μL for pN0 (range 0–20,000 copies/μL); 29,899 copies/μL for pN1 (range 0–488,000 copies/μL), and 118,649.5 copies/μL for pN2 stage (range 0–1,600,000 copies/μL).

### 3.5. Patients with Positive LNs Exhibit Worse Survival Outcome as Determined by Any of the Three Methods

Follow-up data were available for 207 patients (95.4%), with a median follow-up of 33 months (range, <1 to 58.2 months). Thirty-three patients (15.2%) had a local recurrence or distant metastases, of whom 21/33 (63.6%) received adjuvant chemotherapy for having positive LNs on H&E. The median time to relapse was 11.4 months (rate, <1 to 39.5 months), and to death was 22.7 months/range, <1 to 47.8 months). 

**Figure 5:**
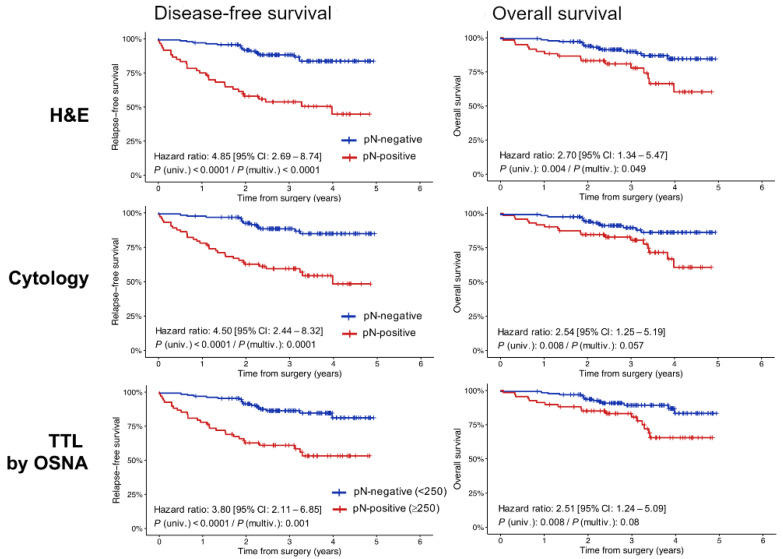
Kaplan-Meier estimates displaying RFS and OS rates according to pN-positive/negative patients by the three methods of LN analysis. *p*-values were calculated using a log-rank test and hazard ratios (HRs) by Cox proportional hazards models both in univariate and multivariable settings. Multivariable models were adjusted by age, sex, pT stage, and histological grade. H&E, haematoxylin, and eosin; TTL, total tumour load; OSNA, one-step nucleic acid amplification; univ., univariate; multiv., multivariable.

We next evaluated the independent prognostic value of having positive LNs analysed by the three methods separately. LN positivity correlated with worse RFS and OS rates for the three methods of LN detection, both at the univariate and multivariable settings (Figure 5; Table 4). Patients with positive LNs detected by cytology smears exhibited significantly shorter RFS and OS, and therefore higher risk of relapse and/or death compared with LN-negative patients (HR: 4.50, *p* < 0.0001 for DFS; and HR: 2.54, *p* = 0.008 for OS). The association with adverse patient survival was also significant for patients with positive LNs when detected by H&E (HR: 4.85, *p* < 0.0001 for DFS; and HR 2.70, *p* = 0.004 for OS). When assessed by OSNA, patients with positive LNs were at a significantly superior risk of cancer recurrence and/or death using a TTL threshold of 250 copies/µL (HR: 3.80, *p* < 0.0001 for RFS; HR 2.51, *p* = 0.02 for OS) (Table 4). As expected in early-stage and locally advanced CRC, the relationship with unfavourable survival outcomes was statistically more pronounced for RFS than for OS (Figure 5; Table 4).

## 4. Discussion

In this large multicentre cohort study, we demonstrated the feasibility of using LN cytology smears for pN assessment in CRC. Moreover, cytology smears were superior in the detection of the presence of tumour cells within the LNs than conventional H&E pathology, with a similar performance to the OSNA molecular assay. We observed that both the number of positive LNs and the positive detection rate, or the proportion of positive patients, were significantly superior when the LN analysis was performed by cytology or OSNA than by H&E (*p* < 0.0001). The high sensitivity of the cytology compared to H&E could be explained by the smooth pressure applied on the LN when performing the circular smear on the slide, which may contribute to squeezing out the epithelial cells from deeper areas of the LN, thus, the cells represented on the smear are not just those from the cut surface.

The concordance between the three methods of LN analysis to classify LN-positive and negative cases was 84.3%. Regarding cytology and H&E, it was very high, with 92.2% of cases (200/217) concordantly classified by both methods of LN analysis. Indeed, the positive predictive value of cytology smears for identifying LN positive cases was 80.3%, and its negative predictive value to differentiate LN negative cases was 98.6% with respect to H&E. In the current study we had 15.7% (34/217) discordant cases between the three methodologies, of which 2.7% (6/217) were patients with positive LNs by OSNA and/or cytology, not detected by H&E. Discordant results were mostly attributed to tumour allocation bias, due that different parts of the LN were used for the different methodologies. 

According to current international guidelines, H&E is the gold standard method of LN assessment and pN stage in CRC. In the pre-screening era of CRC, this method of LN stage has had a good correlation with patient’s recurrence and survival, but it has been demonstrated to be insufficient for the detection of LN micrometastases, which are frequent in early stages of the disease [9]. In fact, stage II CRC patients are still difficult to manage, and 10–15% may recur within 5 years of curative-intended surgery [34]. The latter is attributable to the fact that only a small part of the LN is examined by H&E, which is the reason for requiring the analysis of a minimum of 12 LNs to ensure a reliable pN0 stage [2]. In the setting of pN0 CRC, the presence of LN micrometastases detected by other methods is known to be related to worse survival rates. Several alternative methods of LN assessment have proved to be more sensitive than conventional H&E, allowing for the detection of occult metastases in CRC LNs [9]. Among them, some authors have included both half parts of the LNs in the paraffin block, performing serial H&E sections, or alternating consecutive H&E sections and cytokeratin IHC stains, as it is conducted with sentinel LNs. Other studies have considered the OSNA molecular assay to detect LN metastases with good results [14,24,33,35,36]. In CRC, the OSNA assay has demonstrated its significant superiority to conventional H&E pathology analysis to detect LN metastasis, with an LN upstaging ranging from 10 to 50% [12,15,24,33,36,37,38,39]. In one recent review of 16 studies with OSNA in CRC, the overall diagnostic performance had a specificity of 96.8%, with a concordance rate of 96.0%, and a negative predictive value of 98.6%, confirming the utility of this method of LN analysis [40]. Nevertheless, all the studies performed up to date regarding the analysis of CRC LNs with OSNA, have used one part of the LN for OSNA analysis and the other part for pN staging by means of FFPE and H&E diagnosis [14,22,33]. In consequence, it cannot be disregarded that the current OSNA results published so far, may have an inherent tumour allocation bias of the metastases, and thus, also a bias in the real amount of tumour present in the LNs of CRC. Despite this methodological-related bias, the results obtained with OSNA LN staging in CRC have been encouraging, being better than those obtained with H&E. In this regard, Rakislova et al. first observed that patients with TTL > 7000 copies/µL of CK19 mRNA had an increased recurrence hazard ratio of 4.3. More recently, a study by Itabashi found that OSNA positivity was related to recurrence in stage II CRC patients, with significantly lower 3-year DFS rates (*p* = 0.027). Archilla et al. validated the latter results and observed that a TTL ≥ 6000 copies/µL was related to prognosis with poorer DFS and OS. Thus, TTL has proved to be a prognostic factor related to DFS and OS in CRC [12,15,30]. In addition, the amount of tumour within the LNs, or TTL, is related to the conventional pN stage. Yamamoto observed a progressive increase of the TTL from pN0 to pN2, with 1550 copies/µL for pN0, 24,050 copies/µL for pN1, and 90,600 copies/µL for pN2 patients [24]. These results were endorsed by Archilla et al. with similar TTL values, i.e., 1775 copies/µL in pN0, 49,413 copies/µL in pN1, and 95,000 copies/µL in pN2 cases [15]. In the present study, we also observed that the TTL significantly increased as it increased the pN stage obtained by both cytology and H&E, being under 500 copies/μL for pN0, under 50,000 copies/μL for pN1, and between 110,000 and 144,000 copies/μL for pN2. 

In this study, we observed that LN positivity obtained by any of the three methods of LN analysis was related to worse DFS and OS. Thus, it is advisable to use methods with a high positive detection rate to be able to identify all possible patients with worse outcomes. In addition, there is still an unmet need for a more accurate assessment of LNs in CRC [9]. Importantly, in breast carcinoma, LN analysis with OSNA is performed by using the entire LN tissue, having the TTL both predictive and prognostic values [21,41,42]. LN cytology analysis would not only allow giving an accurate pN stage of CRC patients, but also would enable to use of the whole LN tissue for OSNA analysis, as it is performed in breast carcinoma, and provide complete information of the real tumour burden present within the LN compartment of CRC patients. An alternative new approach to H&E LN analysis should be considered in CRC for an enhanced detection of LN metastases, which would enable direct therapeutic decisions and better patient management [12,15]. 

We performed this large multicentre cohort study to go one stage forward in the CRC LN stage. It represents an intermediate step to using the whole LN for the OSNA assay as it is performed in breast carcinoma. To reach this goal, we analysed CRC LNs by three methods, cytology smears, H&E and OSNA, and compared the pN stage obtained with cytology with the conventional pN stage resulting from H&E, as well as with the OSNA TTL values. We have demonstrated with good results the use of cytology smears as an improved alternative method of the pN stage. Cytology pN stage could be used instead of the conventional pN H&E stage to guide the oncologist in early stages of CRC, as an intermediate step before the use of LN molecular analysis. The next step should be to perform a large study in which the pN stage would be obtained only using cytology smears, plus the analysis of the whole LN with the OSNA assay. The combination of LN cytology smears and the OSNA assay performed on the entire LN tissue would allow to obtain more reliable data of the LN status in CRC. 

A limitation of our study is the inevitable tumour allocation bias, since two parts of the LN were used for the different determinations. Nevertheless, the conventional LN assessment by H&E is far more questionable regarding its reliability to diagnose micrometastases, as only 2–5 microns of the LN tissue are analysed with H&E, and the rest of the LN is either left in the paraffin block or in the formalin. Another limitation is the assessment of the size of LN metastases by cytology smears [43]. Nevertheless, the size of LN metastases in CRC is not as determinant as it is in breast carcinoma since the lymphadenectomy is performed with the colorectal surgical procedure. 

Our results have shown that LN cytology smears are a feasible method for pN staging in CRC, which allows us to integrate the OSNA assay into daily practice pathological diagnosis of early-stage CRC. This procedure would allow for the preservation of a morphology-based pN stage performed with the use of cytology smears, i.e., the number of positive LNs, which meet the current pN stage guidelines, and proceed with the entire molecular LNs analysis. 

## 5. Conclusions

We conclude that cytology-based LN analysis not only enables the performance of the pN stage in CRC but is more accurate and sensitive than H&E to detect LN metastases. It results to have a higher positive patient detection rate than H&E, being comparable to highly sensitive LN molecular analysis. It is a very promising approach and a possible alternative to the conventional pathological pN stage. Using cytology pN stage would enable to use of the whole LN tissue for molecular analysis, which could help to better stage and detect CRC patients at risk of recurrence.

## Figures and Tables

**Table 1:** Clinical and pathological characteristics of the study cohort.

Variable	Strata	Patients (N = 217)	
		*n* %	
Age	Median, years (range)	71 (39–92)	
Sex	Male	129	59.4%
	Female	88	40.6%
Histological grade	High	87	40.1%
	Low	130	59.9%
pT stage	T1	42	19.4%
	T2	61	28.1%
	T3	97	44.7%
	T4	17	7.8%
pN stage by H&E	N0	154	71%
	N1	46	21.2%
	N2	17	7.8%
pN stage by cytology	N0	141	65%
	N1	55	25.3%
	N2	21	9.7%
Total tumor load (CK19 mRNA copies/uL)	Median (range)	19,225.16 (0–1,600,000)	
	<250	145	66.8%
	250 to 6000	15	6.9%
	>6000	57	26.3%
Number of Lymph Nodes (Total: 4310)	Fresh LN	3936	91.32%
	Post-formalin fixation LN	374	8.68%
Lymph nodes (LNs) analysed per patient	Total (median, range)	18 (4–62)	
	Fresh (median, range)	17 (3–62)	
	Post-fixation formol (median, range)	0 (0–27)	
Recurrence	Yes	33	15.2%
	No	184	84.8%
Adjuvant chemotherapy	Yes	55	25.5%
	No	161	74.5%
Lymphovascular invasion	Yes	73	66.4%
	No	144	33.6%
Perineural invasion	Yes	45	20.7%
	No	172	79.3%
Tumour budding	Bd1	120	55.3%
	Bd2	53	24.4%
	Bd3	44	20.3%

**Table 2:** Comparison of the number of positive LN detected by the three methods.

		H&E	Cytology	OSNA
Proportion of Patients/Detection Rate	Number (%)	217 (100%)	217 (100%)	217 (100%)
	Positive LNs	63 (29%)	76 (35%)	72 (33.2%)
	Negative LNs	154 (71%)	141 (65%)	145 (66.8%)
Total number of LNs analysed	Number (%)	3936 (100%)	3936 (100%)	3926 (100%)
	Positive	210 (5.3%)	249 (6.3%)	-
	Negative	3726 (94.7%)	3687 (93.7%)	-
Average number of LNs analysed per patient	Mean (range)	18.1 (3–62)	18.1 (3–62)	-
	Positive	0.97 (0–17)	1.15 (0–17)	-
	Negative	17.2 (1–62)	17 (3–62)	-

H&E, haematoxylin, and eosin; LNs, Lymph nodes.

**Table 3:** Concordance in the pN staging among techniques and diagnostic accuracy.

pN Staging by H&E			pN Staging by Cytology			OSNA		
pN0	pN1a+b	pN2a+b	pN0	pN1a+b	pN2a+b	0 to <250	250 to <6000	≥6000
154 (71%)	46 (21.2%)	17 (7.8%)	141 (65%)	55 (25.3%)	21 (9.7%)	145 (66.8%)	57 (26.3%)	15 (6.9%)
Negative	Positive		Negative	Positive		Negative (<250)	Positive (≥250)	
154 (71%)	63 (29%)		141 (65%)	76 (35%)		145 (66.8%)	72 (33.2%)	
Concordant cases, number (%)			200 (92.2%)			192 (88.5%)		
Discordant cases, number (%)			17 (7.8%)			25 (11.5%)		
Kappa Index			82.1%			73.2%		
McNemar’s *p*-value			0.004			0.11		
Sensitivity (%)			96.8%			87.3%		
Specificity (%)			90.3%			89%		
Positive Predictive Value (%)			80.3%			76.4%		
Negative Predictive Value (%)			98.6%			94.5%		
Number of cases			Cytology-negative	Cytology-positive		OSNA-negative	OSNA-positive	
H&E-negative			139	15		137	17	
H&E-positive			2	61		8	55	

H&E, hematoxylin and eosin; LNs, Lymph nodes.

**Table 4:** Patient’s survival related to lymph node status.

Variable	Strata	Disease-Free Survival (RFS)				Overall Survival (OS)			
		Univariate Analysis		Multivariable Analysis		Univariate Analysis		Multivariable Analysis	
		HR (95% CI)	*p*-Value	HR (95% CI)	*p*-Value	HR (95% CI)	*p*-Value	HR (95% CI)	*p*-Value
pN stage H&E	N1/N2 vs. N0	4.85 (2.69–8.74)	<0.0001	4.27 (2.14–8.49)	<0.0001	2.70 (1.34–5.47)	0.004	2.25 (1–5.08)	0.049
pN stage cytology	N1/N2 vs. N0	4.50 (2.44–8.32)	<0.0001	3.87 (1.95–7.69)	0.0001	2.54 (1.25–5.19)	0.008	2.20 (0.98–4.94)	0.057
TTL	Positive vs. Negative	3.80 (2.11–6.85)	<0.0001	2.90 (1.53–5.50)	0.001	2.51 (1.24–5.09)	0.02	1.97 (0.91–4.26)	0.08

H&E, hematoxylin and eosin; TTL, total tumour load.

## Data Availability

Data are available from the authors upon reasonable request.
